# Distributed Dynamical Computation in Neural Circuits with Propagating Coherent Activity Patterns

**DOI:** 10.1371/journal.pcbi.1000611

**Published:** 2009-12-18

**Authors:** Pulin Gong, Cees van Leeuwen

**Affiliations:** 1 School of Physics, University of Sydney, Sydney, Australia; 2 Sydney Medical School, University of Sydney, Sydney, Australia; 3 Laboratory for Perceptual Dynamics, RIKEN, Brain Science Institute, Saitama, Japan; University College London, United Kingdom

## Abstract

Activity in neural circuits is spatiotemporally organized. Its spatial organization consists of multiple, localized coherent patterns, or patchy clusters. These patterns propagate across the circuits over time. This type of collective behavior has ubiquitously been observed, both in spontaneous activity and evoked responses; its function, however, has remained unclear. We construct a spatially extended, spiking neural circuit that generates emergent spatiotemporal activity patterns, thereby capturing some of the complexities of the patterns observed empirically. We elucidate what kind of fundamental function these patterns can serve by showing how they process information. As self-sustained objects, localized coherent patterns can signal information by propagating across the neural circuit. Computational operations occur when these emergent patterns interact, or collide with each other. The ongoing behaviors of these patterns naturally embody both distributed, parallel computation and cascaded logical operations. Such distributed computations enable the system to work in an inherently flexible and efficient way. Our work leads us to propose that propagating coherent activity patterns are the underlying primitives with which neural circuits carry out distributed dynamical computation.

## Author Summary

The brain processes information with extraordinary efficiency, and can perform feats such as effortlessly recognizing objects from among thousands of possibilities within a fraction of a second. This is accomplished because the brain represents and processes information in a distributed fashion and in a dynamical way. This processing is manifested in spatiotemporal neural activity patterns of great complexities within the brain. Here, we construct a spiking neural circuit that can reproduce some of the complexities, which are evident in terms of multiple wave patterns with interactions between each other. We show that their dynamics can support propagating pattern-based computation; spiking wave patterns signal information by propagating across neural circuits, and computational operations occur when they collide with each other. Such dynamical computation contrasts sharply with that done by static and physically fixed logic gates operating in other computing machines such as computers. Moreover, we elucidate that the collective dynamics of multiple, interacting wave patterns enable computation processing implemented in a fundamentally distributed and parallel manner in the neural circuit.

## Introduction

To understand brain function, it is essential to study the collective electrical activity of neural circuits [1]. This activity typically exhibits intriguing spatiotemporally organized patterns: they are commonly observed in multi-unit electrophysiological recording, EEG local field potential recording, MEG, optical imaging and fMRI imaging, both in spontaneous activity [2]–[5] and evoked responses [6]–[21]. In space, these patterns often take the form of localized patches or clusters of activity [2]–[16]. Recordings over large populations of neurons have shown that several of such localized patterns can occur simultaneously across cortical regions [2]–[16]. Over time, these patterns often do not remain at specific locations. As self-sustained entities, they propagate or move about in space [4]–[8], [10]–[16]. In doing so, they interact with each other, resulting in dynamical collective behavior. Here we will consider what kind of functional role this behavior may have.

Propagating coherent patterns have been registered in the experimental literature as “spreading” or “drifting” activity [4]–[8] or as “traveling waves” [13]–[24]. The simultaneous presence of several of these patterns has been observed in the spontaneous activity of cat visual cortex [4],[5; see 25 for a corresponding model study], in evoked response patterns in turtle olfactory bulb [14], and visual cortex of various species [9],[15], as well as in sensorimotor cortex of behaving mice [7]. When several localized, moving patterns occur together, they are likely to interact. Indeed, interactions have been shown to occur in rat somatosensory cortex [13]. To describe the collective activity in olfactory, visual, auditory and somatosensory cortices of behaving rabbits, the term “interacting wave packets” was explicitly used [11],[12], which nicely captures the relevance of propagations and interactions of these patterns.

Despite the ubiquity of these patterns and their interactions, their fundamental functional role has remained unknown. Although some authors have speculated on the role of propagating waves [26], the functional implications of other aspects such as the simultaneous presence of multiple propagating patterns or their interactions have remained completely unclear. Current theoretical frameworks describe neural activity either in computational or dynamical systems perspectives. Conventional computational theory is based on the manipulation and representation of static symbols [27]. This perspective contradicts the temporal variability of brain activity, which calls for a dynamical systems approach. When dynamical systems theories are applied to neuroscience, the prevailing concept is that of stable low-dimensional attractors [28]. This notion, although it has provided many important insights, is less suitable to capture the functional role of brain activity in its actual spatiotemporal complexity.

We need to resolve the restrictions of conventional computation and standard dynamical systems theories, in order to describe neural activity and understand its fundamental function. This study is based on the consideration that neural circuits are spatially-extended, pattern-forming systems, containing large numbers of simple neurons with spatially restricted connectivity [29],[30],[31]. In spatially extended physical systems composed of large numbers of simple interacting elements, such as reaction-diffusion systems and fluidic systems, localized propagating coherent patterns are a common feature known under different names, including wave packets, spots, breathers and soliton waves, amongst others [32],[33],[34]. They are an emergent, collective property of these systems.

Using these systems as analogy, we construct a simple, spatially extended neural circuit model to represent the gross architecture within the cerebral cortex. As an emergent, collective property of the system, the circuit exhibits dynamical activity patterns, reproducing some of the complexities observed in empirical studies. In particular, the circuit provides simultaneous propagation of multiple locally coherent patterns and their interactions. By revealing how their ongoing collective behavior can naturally embody computation, we demonstrate what fundamental function these patterns can serve.

Propagating coherent spiking patterns can support several essential aspects of a computational processing. As self-sustained objects, these patterns can signal information by propagating across neural circuits. Information processing, or computation, occurs when they interact or, specifically, collide with each other. Collectively, these patterns perform distributed, parallel and cascaded computational operations, thereby enabling neural systems to work in an efficient and flexible way. We shall call this *distributed dynamical computation*, which is proposed as a framework for understanding spatiotemporal propagating activity patterns in neural circuits. This understanding links their dynamics with a form of non-conventional, abstract computation.

## Results

### 

#### Qualitative characterization of spatiotemporal patterns

Significant correlations exist between neural activities recorded at different levels, from spikes and field potentials to fMRI [4],[35]. We focus this study on the most basic of these levels: neuron spiking behavior. Given that the myriad details of neuronal anatomy and its function are only partially known, rather than modeling neurons in great detail, we use a simple model (see Methods section) in an effort to capture some of the features of real neurons and neural circuits crucial for dynamical spatiotemporal pattern formation.

Starting from random initial conditions and after initial transients, the neural circuit generates collective activity, in which localized pattern structures with temporal regularities are clearly in evidence (see Methods section for more details including coherent and incoherent spiking patterns). By varying the excitatory coupling strengths 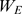 and inhibitory coupling strengths 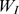 (See Methods section) within the range considered, we distinguish three types of patterns. The patterns are qualitatively characterized by the following phenomena, respectively: (1) localized incoherent spiking patterns occur, which slightly move around; the motions are constrained within local areas without any long-range movements; (2) several spatially localized coherent patterns move about; the movements are long-range across space and over time; there are many interactions or collisions between them, resulting in complex, dynamical collective behaviors; (3) several localized coherent patterns occur, showing regular motions without complicated interactions.

**Figure 1:**
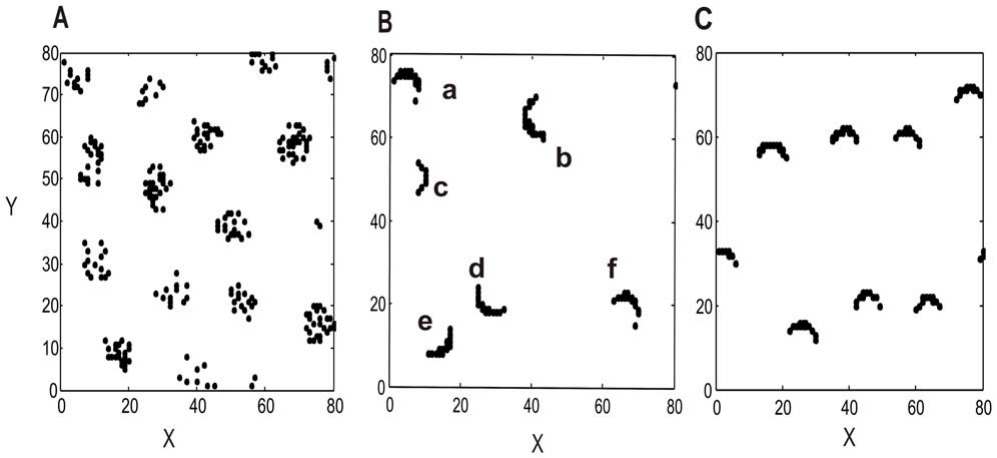
Snapshots of three different types of spatiotemporal patterns distinguished in the circuit model. Each neuron is oscillatory with a frequency of 11Hz. Black dots indicate the coordinates where neurons are firing. (*A*) Type 1 (with parameters 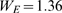, and 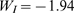): spatially localized patterns that lightly jitter around; (*B*) Type 2 (with parameters 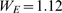 and 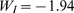): localized coherent patterns with long-range movements and complicated interactions. Each pattern is labeled by a distinct letter. At this time moment, the system has five spatially localized structures labeled from a to f, of which the center-of-mass positions are respectively: *(5.1, 74.2), (39.7, 64.2), (9.3, 50.5), (27.3, 19.7), (15.1, 9.9), (67.2, 20.8)*. (*C*) Type 3 (with parameters 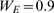 , and 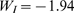): localized coherent structures with regular motion and regular overall features.

**Figure 2:**
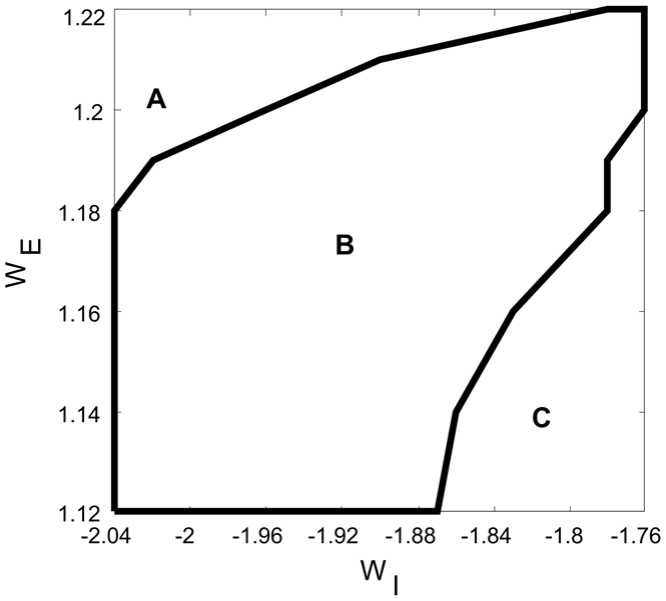
Phase diagram of spatiotemporal activity patterns in the 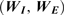 parameter space. The region B circled by the dark line is the region in which the system generates the Type 2 patterns, the region A for Type 1 patterns, and the region C for Type 3 patterns.

Fig. 1 shows the instantaneous activity patterns of each type and Fig. 2 maps out where these three types of patterns occur in the parameter space. Among these patterns, those of Type 2 appear the most intriguing ones, showing the greatest space-time complexity in their overall behavior. Several coherent patterns originate at apparently random locations, and propagate in all possible directions. Each time a pattern sweeps through a given region, the direction varies. Sometimes patterns are spontaneously annihilated, but most of time they persist, traveling over long distances as self-sustained objects. These distances for the most part exceed the coupling ranges and can cover the whole space of the model circuit. The patterns interact when they meet; this includes non-destructive interactions, in which the moving patterns modulate each other's states, as well as destructive ones, in which one or both of the patterns are annihilated.

Patterns of Type 2 qualitatively reflect many of the features observed in empirical studies, particularly the distributed properties of multiple activity patterns [4]–[7], [11]–[16], their propagations [4],[5],[11],[12],[14],[15], and their interactions [11],[12],[13]. In addition, the movements have a seemingly random feature. Several experimental studies, for instance in cortical local field potentials of rabbits, have pointed out that localized coherent structures called “wave packets” originate from random locations and propagate in variable directions [11],[12]. Propagating coherent activity patterns termed “traveling waves” have the likewise variability of moving speeds and directions in the collective activity of monkey and cat visual cortex and in that of rat hippocampus [22],[23].

#### Quantitative properties of dynamical spatiotemporal patterns

**Figure 3:**
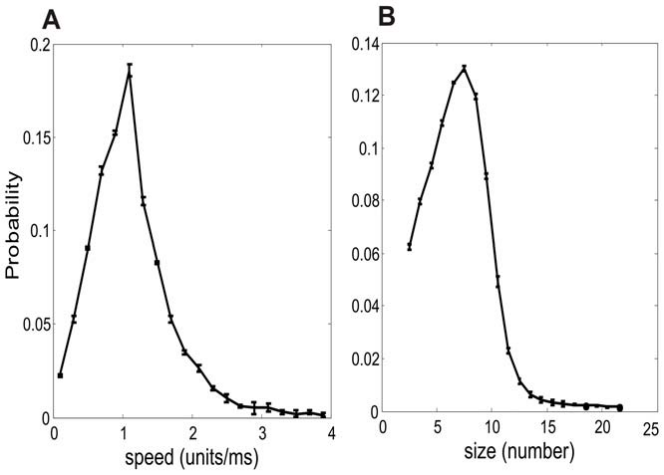
Statistical properties of the propagating coherent spiking patterns. (A) The distribution of the propagating speeds of the dynamical patterns; (B) the distribution of the patterns' sizes.

Since the collective behavior of Type 2 reflects some key features of activity patterns observed in real neural circuits, we shall mainly focus on these patterns and investigate their quantitative properties. As propagations are the most obvious dynamical feature of these patterns, a convenient quantitative measure is their velocities. As shown in Fig. 1*B*, collective activity at any moment is sustained by the clusters of neurons. We label each of them with a letter. We calculate the center-of-mass position 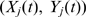 of *j*th cluster at time moment *t*, 
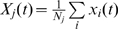
, 
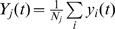
, where 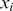 and 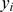 are the *x* and *y* position of *i*th neuron of the cluster, and 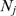 is the total number of neurons in the cluster. We define 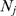 as the size of the cluster. For Pattern ***a*** in Fig. 1*B*, its center-of-mass is positioned at *(5.1, 74.2)*. Based on center-of-mass positions, a *2*-dimensional velocity with components in *x* and *y* directions is: 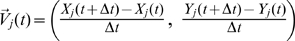. The magnitude of velocity is speed 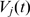: 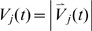. We consider the velocity with 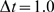 ms. Table 1 gives the velocities of the localized patterns shown in Fig. 1*B*. To get further quantitative characteristics of these patterns, we calculated the distributions of their sizes and their speeds, which are shown in Fig. 3. It is interesting to note that the distributions are qualitatively similar to that of traveling waves in rat hippocampus [24] and cat visual cortex [22]. In addition, to understand how these patterns change as the parameters change when the system is in the regime of Type 2 patterns, we have calculated the change of the mean values of the speeds and sizes as a function of system parameters. The results are shown in Fig. S1 and Fig. S2 in the supporting information.

**Table 1:** The velocities of the spatially localized patterns.

Localized pattern	a	b	c	d	e	f
Velocity	(0.6, 2.0)	(−1.0, −1.6)	(1.0, 0.0)	(1.2, −2.4)	(1.2, −1.2)	(0.7, 1.1)

Another significant property of Type 2 patterns involves the complex dynamics of their collective propagating behavior, which can be quantified by mean-squared-displacement (MSD). Firstly, for each localized activity pattern, its traveling trajectory is obtained by feeding its center-of-mass positions into an algorithm developed for tracking them over time. Based on the trajectories of all moving patterns, the MSD as a function of time increment 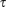 is:
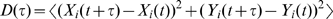
(1)


The bracket 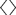 represents averaging over time *t* and across trajectories. On a given trajectory, 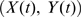 and 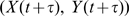 are its positions at time moments *t* and 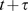.

**Figure 4:**
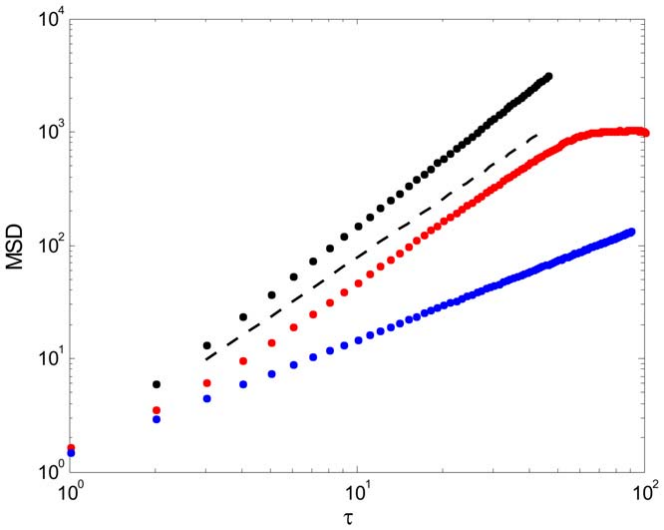
Log-log plot of mean-squared-displacement (MSD) as a function of time increment for different collective motions. The red dots, representing the coherent patterns of Type 2, show a clear straight-line part in the log-log plot, fitting to an exponent of *1.73* (dashed line). The cut-off is due to the finite-size of the circuit. For the black dots, representing Type 3 patterns, the exponent of the fitted line is *2.0*. The blue dots represent random Brownian motion, corresponding to an exponent of *1.0*.

We calculated the MSD for Types 2 and 3 patterns, since they involve long-range propagations across the circuit. Fig. 4 (red dots) shows the log-log plot of the MSD as a function of 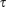 for Type 2 patterns. As the plot shows, the MSD function appears to follow a straight line, suggesting that it is a power function of the time increment. To verify this observation, we used the maximum-likelihood method [36], and obtained the result that the best fit is a power function, 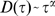 with an exponent 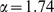. With different system parameters, the exponent for the patterns of Type 2 is in the range 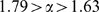. Type 3 patterns, shown in black dots in Fig. 4, have a MSD with exponent 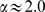, which characterizes regular movements along straight lines. For comparison, we also show in blue dots a normal random diffusion process (Brownian motion), for which the scaling exponent is 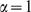. A MSD as a power function of 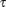 and with an exponent larger than 1 and smaller than 2 indicates that the collective propagating behavior is neither a fully random motion nor a regular motion, instead it is in-between these two extremes. In fact, the collective behavior of Type 2 patterns is a kind of non-normal diffusion process, known as anomalous super-diffusion [37],[38].

The fact that the behavior of Type 2 patterns belongs to the class of non-normal diffusion process is quite informative; it indicates that there are long-range spatiotemporal correlations for the propagating patterns [37],[38]. In neuroscience, nontrivial spatial and temporal correlations have been very well documented by analyzing brain activity from several different perspectives, including the temporal fluctuations of power of brain oscillations [39],[40], the distribution of size of neural avalanches [41], and the intervals of synchronized activity [42]. Our present study provides an alternative measure of nontrivial spatiotemporal correlations, regarding specifically the characteristics of propagating wave patterns that have been ubiquitously observed in brain activity.

The above described quantitative measures reveal the main features of Type 2 patterns. These measures can therefore be used to obtain a parameterization scheme for the present model, in order to show the existence of the dynamical patterns in the parameter space, as was done in obtaining Fig. 2. Our explorations of the model with much larger numbers, such as 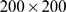, of neurons have suggested that Type 2 patterns are quite common. For instance, if 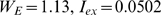, a model with 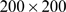 neurons shows Type 2 patterns when 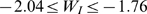.

#### Dynamical computation by propagating coherent activity patterns

Having characterized the complexity of the propagating patterns, we are now ready to approach the question of their fundamental function. This question can now be specified: how do the dynamical patterns enable the system to do computation? To develop answers to this question, we use the methodology of examining how general-purpose computations can be embedded in autonomous dynamical processes without setting up specific computational tasks. Note that because of its conceptual simplicity and convenience, the methodology of revealing general-purpose computation based on the autonomous dynamics of a system has played an important role in developing a computational theory of the brain [43] and investigating the general computational capabilities of dynamical systems [44].

**Figure 5:**
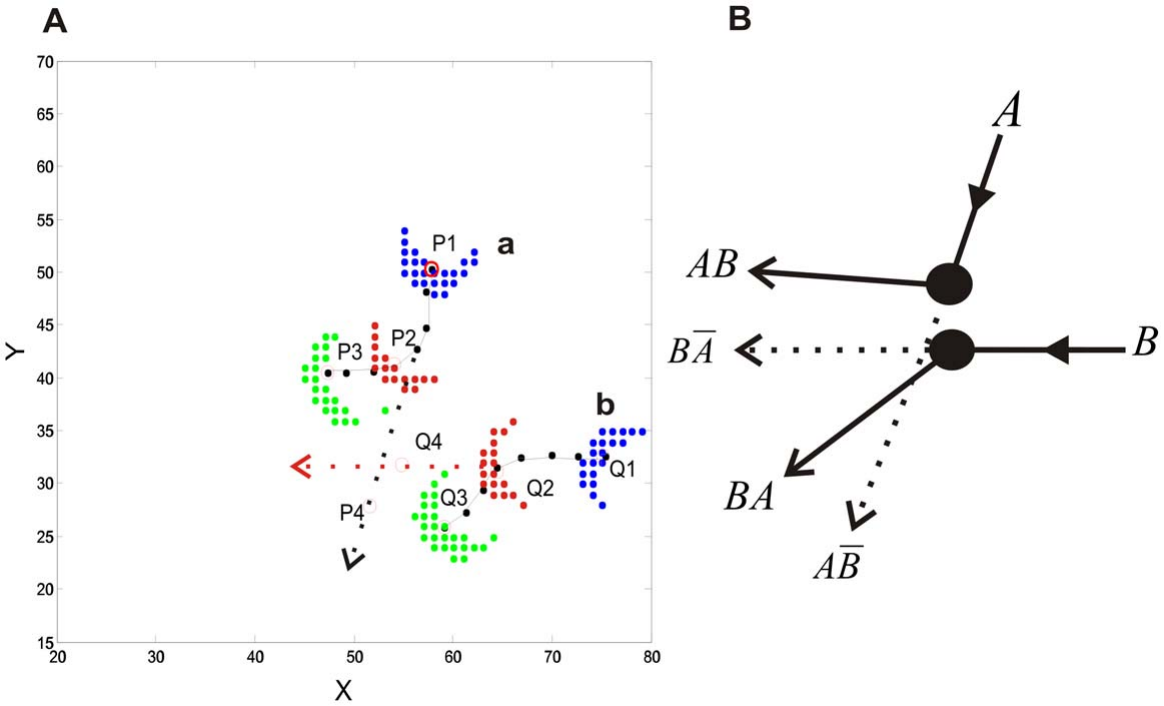
The space-time behavior of two, moving localized coherent patterns and the dynamical logical operations based on the interaction between them. (*A*) Among several other activity patterns, two of them are shown here. Different colors indicate the patterns at different time moments: blue at *t1 = 620503* ms, red at *t_2_ = 620507* ms, and green at *t_2_ = 620510* ms. At *t_1_*, Pattern *a* is centered at position *P1 (57.8, 50.4)* and Pattern *b* at *Q1 (75.1, 32.6)*. Time *t_2_*, is the moment when the collision between the two moving patterns happens. At that time, Pattern *a* is at *P2 (54.0, 41.1)* and Pattern *b* at *Q2 (64.4, 31.6)*. Afterwards, at *t_3_* Pattern *a* is at *P3 (47.3, 39.5)* and Pattern *b* at *Q3 (59.0, 25.9)*. Without a mutual collision, Pattern *a* would have traveled along the dashed black line and passed through *P4* at time moment *t_3_*, and Pattern *b* along the dashed red line and through *Q4* at *t_3_*. To see the positions *P1*, *Q1*, *P3*, *Q3*, *P4*, and *Q4* clearly, they are also noted by red circles. *(B)* An illustration of interaction-based logical operations. The two filled black circle are used to represent patterns at the time moment when they collide with each other. Arrows correspond to their trajectories prior to and after the collision; dotted arrows correspond to trajectories that occur if the other signal is absent. The signal *AB* represents ‘*A* AND *B*’, and 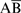 represents ‘*A* AND NOT *B*’. *A*, *B*, *AB*, *BA*, 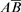 and 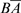 are corresponding signals at the positions *P1*, *Q1*, *P3*, *Q3*, *P4*, and *Q4*.

For these purposes, let us consider that at any time there are several spatially localized coherent patterns. These can be labeled by letters as demonstrated in Fig. 1*B*. From among several moving patterns, we select two of them, Pattern *a* and Pattern *b*, without loss of generality, at an arbitrary time moment after an initial transient, 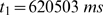. We then focus on the way they propagate and interact. The detailed space-time behavior of the two coherent patterns can be viewed in Video S1 in the supporting information, which we recapitulate in Fig. 5*A*. At time moment 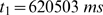, Pattern *a* is centered at position *P1 (57.8, 50.4)*, and Pattern *b* at position *Q1 (75.1, 32.6)*. The two patterns propagate along their own paths, represented as black lines in Fig. 5A until around time moment 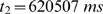, when Pattern *a* is at *P2 (54.0, 41.1)*, and Pattern *b* is at *Q2 (64.4, 31.6)*, where they collide with each other. After that, their states and therefore their momentums are changed compared to those before their collision. Here “collision” is used to describe a co-current change in two structures' momentums due to their physical proximity, even though the objects do not actually touch each other. Because of the lateral inhibitory coupling, each moving pattern gets inhibitory effects from another one when they propagate to approach each other, hence resulting in the repulsive interactions as shown in Fig.5A. After the collision at time moment 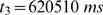, there are coherent structures centered at positions *P3 (47.3, 39.5)* and *Q3 (59.0, 25.9)*.

In order to examine how abstract computational operations can be embedded in the ongoing interactions between propagating coherent activity patterns, a simple manipulation can be helpful. For instance, consider the situation before the collision, at a time moment such as *t_1_ = 620503 m*s or at any other time along the incoming path. If we eliminate all the spikes belonging to Pattern *b*, there will be no encounter at the rendezvous point *Q2 (64.4, 31.6)* at time moment *620507 ms*; Pattern *a* will continue, unaffected by Pattern *b*, to move in the same direction. Thus it will follow the path indicated by the dashed black line in Fig. 5*A* to pass through *P4 (51.6, 27.9)* at time moment 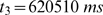. Similarly, if all spikes in Pattern *a* are eliminated at *t_1_*, Pattern *b* will continue its original path along the dashed red line and pass through *Q4 (54.8, 30.1)* at 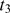. These results demonstrate that two moving spiking patterns effectively modulate each other when they meet at the right time and at the right place.

To reveal how the collective behaviors of these patterns can support the essential aspects of a computational processing, which include signal (or “information”) transmission and signal (or “information”) processing [45], their dynamics is considered at a more abstract, computational level of analysis. Firstly, we perform the following abstraction: the presence of a localized coherent activity pattern at a particular position within the circuit signifies ‘1’, whereas its absence signifies ‘0’. Hence, based on the abstraction, a localized coherent pattern that is propagating can represent a *bit of information (or “signal”)*. It is in the spirit of McCulloch and Pitts' classical study to abstract from neuronal activities to binary values in order to develop a computation theory of the brain [43]. In [43], neural activities are excitatory and inhibitory synaptic inputs of threshold neurons along fixed lines, while here the relevant activities are the coherent patterns at the level of spiking neural circuits and are emergent properties unconstrained by fixed lines. Because the presence of a localized pattern is abstracted or interpreted to represent a bit of information ‘1’, through its dynamical behavior, i.e., its propagation, the bit of information (or the signal ‘1’ ) can then be transferred along its propagating path from one part of the circuit to another. For an illustration, when the Pattern **a** propagates along its path from *P1 (57.8, 50.4) to P2 (54.0, 41.1)* shown in Fig. 5A, a bit of ‘1’ signal is transferred along this path from position *P1 to P2*, and clearly the speed of the information transfer is the propagating speed of the pattern. Thus, the propagating behavior of the coherent patterns is a primary mechanism for transferring information over long space-time distance. Interestingly, the functional role of the propagating behavior makes its trajectories analogous to real physical wires used to transfer electrical pulses in electrical circuits.

Thus far, we have introduced the emergent localized coherent patterns with their role of representing signals, and elucidated that their propagation can support an essential function of a computational processing, which is signal transmission. We shall now turn to signal or information processing. The processing can be embedded in the dynamical interactions between these patterns: the patterns implement logical functions and the locations of their possible encounters act as logic gates. This principle is illustrated schematically in Fig. 5*B*, in which moving spiking patterns are represented by filled circles for an illustrative purpose.

In Fig. 5*B*, signals **A** and **B** represent, respectively, the presence or absence at 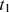 of localized activity patterns at positions *P1* and *Q1* shown in Fig. 5*A*. The interaction logic gate at *P2*, *Q2* can carry out the following operations: **A** AND **B** or, equivalently, **B** AND **A**, **A** AND NOT **B**, **B** AND NOT **A**. At time moment 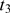, 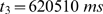, there will be a coherent pattern at position *P3* if there was one at *P1* and another at *Q1*. No other patterns are involved in these particular interactions. Hence, signal **C** at *P3* is ‘1’ if and only if **A** is ‘1’ and **B** is ‘1’. So we have: **C** = **A** AND **B**. Likewise, signal **D** at *Q3* is ‘1’ when both B and A are ‘1’ (**D** = **B** AND **A**), realizing the same AND function. Similarly, there will be a pattern at *P4* if and only if there was one at *P1* and none at *Q1*. The signal **E** at position *P4* is: **E** = **A** AND NOT **B**. Signal **F** at position is *Q4*: **F** = **B** AND NOT **A**, which implements the same AND NOT function. Owing to its AND and NOT capabilities, the interaction gate is a universal logic primitive. By abstractly depicting the localized coherent patterns as bits of information, signal processing or computation can be accomplished in terms of logical functions. Hence, the dynamics of the propagating patterns, that is, their collisions, can support another essential function of a computational processing, which is signal processing. The logical operations performed here are reminiscent of those done with collisions in the billiard ball model [45],[46],[47], which has played a very important role in linking basic physical laws with computation theory.

Considering the number of neurons active within a cluster as its ‘mass’ and using its propagation velocity, we can calculate whether the collision preserves momentum. The velocity of Pattern a before collision is 
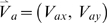
 = *(−1.26, −1.69)*, and that after collision is 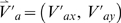 *= (−2, −0.4)*. The size or the mass of Pattern a is 12, that is, 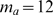. For Pattern b, its velocity before collision is 
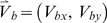
 = *(−2.45, −0.9)* and that after collision is 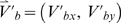 *= (−1.35, −2.15)*. The mass of this pattern is 13, 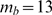. The *x*-component of total momentum before collision and after collision is 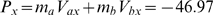 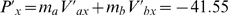, respectively. Apparently 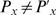, thus there is no conservation of momentum; the interaction is a kind of inelastic collision; and so it is not reversible. This is generally the case for interactions in our model. In this respect, our model differs from the billiard ball model [46],[47],[45], in which interactions are typically reversible.

#### Cascaded computational operations

**Figure 6:**
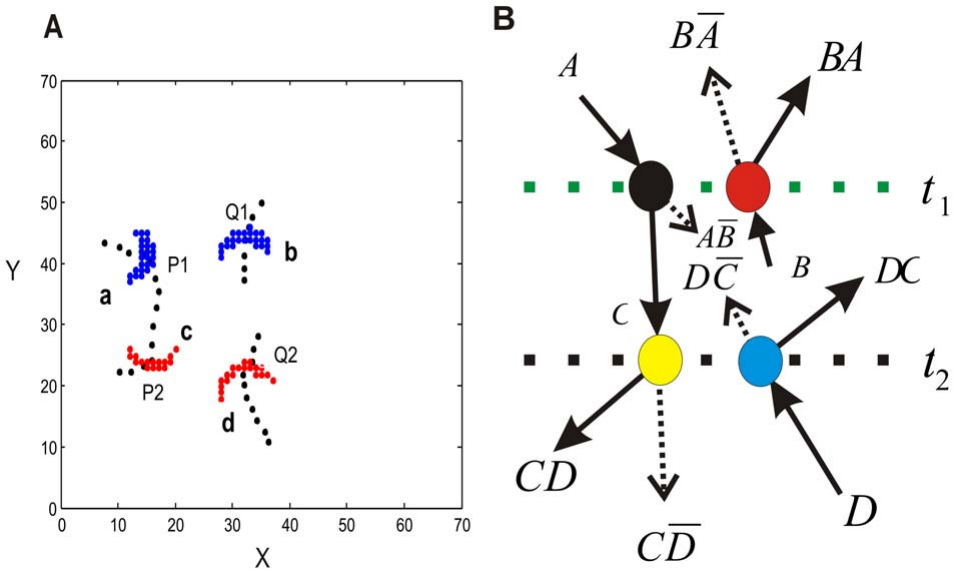
The space-time behavior of several localized coherent patterns and cascaded computational operations. (*A*) At time 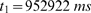, the two coherent Patterns *a* and *b* depicted in blue collide with each other. The output is a coherent Pattern *c* (red) which, at time 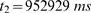 is positioned at *P2*, where it collides with Pattern *d* coming from a different direction. The dashed black lines show the trajectories of the propagating patterns. (*B*) Illustration of the cascaded logical operations. *A*, *B*, *C*, *D* are signals signifying the presence or absence of coherent activity patterns at time moments *t_1_* and *t_2_*. Based on these signals, the logical operation occurring at *t_1_* is located on the green dotted line, and the one at *t_2_* is located on the black dotted line. The output signal from the operation at *t_1_* that involves *A* and *B* is a signal *C*, *C* = *A* AND *B*, which acts as the input signal for the operation happening at *t_2_*, which also involves a signal *D*. The dashed arrows correspond to the situation that one of these signals is absent.

The elementary logical operations demonstrated above can essentially be interpreted as computational building blocks in neural circuits. To show this, we need to demonstrate that they are cascadable, that is, the output of one logical operation is able to be used as an input to another one [48]. In other words, these operations must support compositionality. For the elementary computation as shown in Fig. 5B, both input and output signals are propagating activity patterns. Indeed, output of one operation can later be used as input for another one. During the ongoing evolution of the activity patterns, as shown in Fig. 6*A* and the corresponding Video S2 in the supporting information, at 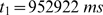 an interaction happens when Pattern *a* is at *P1 (14.3, 41.4)*, and Pattern *b* is at *Q1 (32.0, 44.0)*. The outcome of this interaction is carried through space by propagating patterns, one of which at 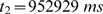 is located at *P2 (15.9, 24.2)*, where it interacts with another one, Pattern *d* at *Q2 (31.7, 21.9)*, which comes from a different part of the circuit. Note that only those patterns relevant for illustrating the cascaded operation are shown in Fig. 6*A*. The example shows how populations of neurons collectively route signals through the circuit in a manner that naturally embeds cascaded operations.

The cascaded logical operations are shown in Fig. 6*B* in an abstract form. Particularly, we can get the composed outputs, such as **A** AND **B** AND **D**. The occurrence of the computation represented by collision of the yellow and blue filled circles at Positions *P2 (15.9, 24.2)* and *Q2 (31.7, 21.9)* at time moment 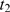 is enabled by the computation that has previously occurred at time moment 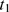. During the cascaded operations, propagation is essential to make local information available at larger spatial scales and to assemble signals that are distributed over space and time.

#### Distributed parallel computational processing

**Figure 7:**
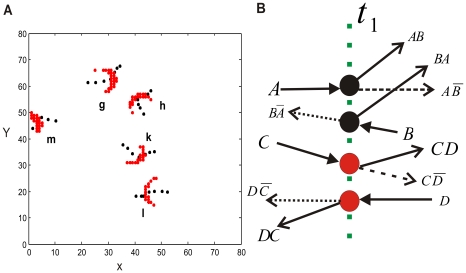
The space-time behavior of propagating, coherent activity patterns and corresponding parallel computations. (A) At time moment *t = 862039* ms, two collisions happen simultaneously. Pattern *g* collides with Pattern *h*, and Pattern *k* collides with Pattern *l*. The dashed lines are the trajectories of the five moving coherent patterns. (B) Illustration of interaction-based parallel logical operations. *A*, *B*, *C* and *D* are input signals. The vertical green dashed line indicates where two computations happen at the same time moment 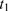, 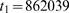 ms. The two black filled circles represent a pair of signals involved in one logical operation, and the two red filled circles represent the signals in another one. Each of these operations can produce ‘AND’, ‘AND NOT’ functions. The dashed arrows correspond to the situation that one of these signals is absent.

At the computational level of analysis, the pattern dynamics supports two fundamental activities of a computational processing: signal transmission and signal processing. We now turn to the functional implications of multiple, propagating patterns that are distributed over the different parts of the circuit. Indeed, the simultaneous presence of these patterns and their ongoing behavior can provide the needed substrate for distributed parallel signal transferring and processing. Fig. 7*A* (Video S3 in the supporting information), shows that, Pattern *g* and Pattern *h* collide with each other at time moment *t = 862039 ms*, when *g* is at *(30.7, 62.7)* and *h* is at *(41.0, 55.1)*. Meanwhile in the other parts of the circuit the Pattern *k* and Pattern *l* collide, when *k* is at *(41.6, 33.2)* and *l* is at *(45.2, 19.9)*. This example illustrates that several interactions can occur in parallel at the different parts of the circuit. The parallel interactions embody the parallel logical operations shown in Fig. 7*B*. They involve the Patterns *g–h* and *k–l*, which are physically distributed over the circuit. Furthermore, another localized pattern such as Pattern *m*, located at a different position, is moving along its own path. This one, as we have shown above, has the function to transfer a binary signal ‘1’ along its path. Thus, propagating patterns co-occurring can at any time either transfer signals or process signals, resulting in a computational processing carried out in a distributed parallel way.

**Figure 8:**
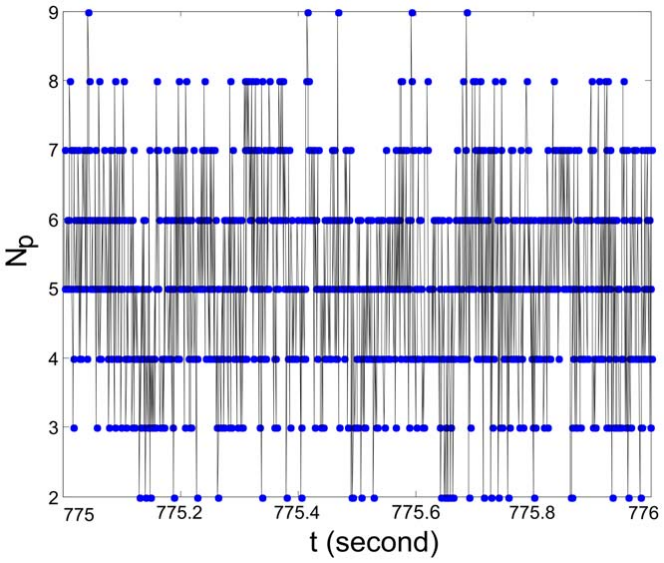
The number of co-occurring localized, coherent spiking patterns as a function of time, with the parameters 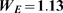, 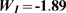.

It is important to emphasize that the distributed computing scheme in the spatially-extended spiking neural circuit exhibits the typical features of the parallel distributed processing paradigm proposed for the brain: a set of large number of neurons, recurrent connections without central controllers, and patterns of activation distributed across neurons [49]. In our case, however, these patterns consist of spiking activity collectively propagating. Distributed computational processing is supported by the co-occurrence of several of these patterns; each of these patterns either propagates along, or collides with others. Based on this consideration, the number of co-occurring patterns, to some degree of approximation, can be used to estimate the system's distributed parallel processing capacity. Some factors, such as the range of coupling would delimit the maximal number of coherent structures operating simultaneously in the system. When the system is in the Type 2 regime, generally more patterns propagating simultaneously will result in more collisions. For our current model, we calculated the number of co-occurring localized coherent patterns, which is denoted as 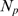 at each time moment to characterize the complexity of the distributed computational processing. The result is shown in Fig. 8. Note that at any time moment considerable numbers of patterns are involved in carrying out the different aspects of a computational processing: signal transmission (propagations without collisions) or signal processing (collisions).

#### The effects of external perturbations

**Figure 9:**
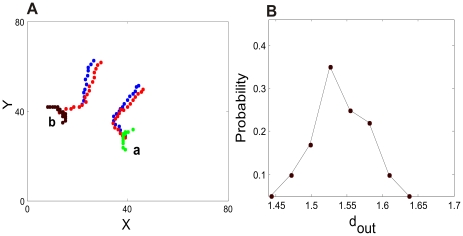
The space-time behavior of propagating coherent patterns with and without external perturbations. (A) The blue dots are original propagating trajectories of Pattern *a* (green color) and Pattern *b* (black color) without external perturbations. The two patterns collide when Pattern *a* is located at (34.35, 35.86) and Pattern *b* is at (22.4, 44.8) respectively. Before the collision, Pattern *a* is located at (38.9, 29.4) and Pattern *b* is located at (13.1, 39.2). The red dots are propagating trajectories of these two patterns after external perturbations. (B) The distribution of distances between the perturbed outgoing trajectories after collisions and the corresponding original ones.

We have studied how general-purpose computation is implemented based on ongoing, autonomous dynamics of the propagating patterns. A question that naturally arises is: how does the system deal with external perturbations? To answer this question, firstly, we add external perturbations to one of ongoing propagating patterns. We then follow the evolution of both the perturbed and the unperturbed systems in order to capture the spreading of the perturbations. For instance, for Pattern *a* shown in Fig. 9*A*, in the original system, the pattern moves along the black line and it interacts with Pattern *b* when it reaches the position *(34.4, 35.9)*. After external perturbations are added to Pattern *a*, as shown in Fig. 9*A*, the propagating trajectory of the pattern (red line in Fig. 9*A*) is slightly shifted in comparison with the original propagating path. This consequently results in the situation that Patterns *a* and *b* collide at a slightly different time moment at a slightly different position, compared to the original event. This shift leads to a different outgoing propagating trajectory (the trajectory after collision) for Pattern *b*. It is important to notice that before the collision, there are no changes to the propagating path of Pattern *b*; it is just the collision between the perturbed propagating pattern with an co-occurring one that results in the changes in the outgoing path of Pattern *b*. Instead of interrupting ongoing activity patterns, external perturbations modulate the propagating trajectories and interactions.

To characterize the effects of external perturbations, we calculated the distance between the outgoing trajectories when there are external perturbations and the corresponding original outgoing ones. The distance is defined as: 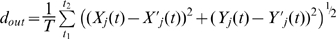, where 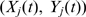 is the center-of-mass position of a pattern of the original system and 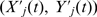 is that of the corresponding pattern of the perturbed system. 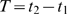, is total time length considered for an outgoing trajectory after a collision. In the current study, *T = 8 ms*. In order to obtain the statistics of the modulation, external perturbations are added to many different interacting pairs of propagating patterns before their collisions. Fig. 9*B* shows the distribution of the distance. We have also calculated the dynamics of the perturbed propagating trajectories and found that they also can be quantified as an anomalous super-diffusion process. This indicates that the statistical properties of the collective motions of the propagating patterns are preserved under external perturbations. Let us note that experimental studies have found that, in the visual cortex of freely viewing ferrets, stimulus-evoked activity reflects the modulation and triggering of intrinsic circuit dynamics by sensory signals, with a preservation of collective correlations of neural firing rates [50].

## Discussion

The importance of spatiotemporal dynamical patterns in the brain has been proposed in [29], with an emphasis on spatial modes and their coupling. Here, we have focused on propagating coherent activity patterns, which are ubiquitous in the brain. These dynamical patterns are neither random nor stable; rather they are characterized by rich dynamical behaviors. We have used a simple, stereotypical spiking neural circuit to generate spatially localized propagating patterns. The patterns capture some of the key features of real pattern complexities: a distribution of multiple localized activity patterns, their propagations and their mutual interactions. To understand their fundamental functional role, we propose the notion of distributed dynamical computation. Localized propagating patterns are the underling primitives of dynamical computation; over time they transfer information across space and process information through their interactions. Collisions distributed over different locations and occurring at different time moments can be connected to each other by propagating patterns. This mechanism enables elementary computations to occur in a cascaded fashion, resulting in more complex computations. In addition, several interactions distributed across different locations can occur simultaneously, resulting in parallel processing.

Dynamical computation emerges on the basis of activity in neural circuits; they enable and sustain propagating localized patterns and their interactions. In this framework, the propagation and processing of signals are fluid; signals do not rely on the fixed physical lines of neural circuits to guide their propagation trajectories. The computations are not confined to specific anatomical sites; rather they occur wherever moving patterns collide with each other. With respect to real-brain architecture, this is clearly a simplification. We may consider the neural architecture as biasing the trajectories of propagating patterns to various extents. Nevertheless, a certain independence of fixed connectivity structure must be at the basis of how flexibility in brain functions is achieved.

Propagating coherent activity patterns implement logical operations in a manner reminiscent of the collisions in the classical billiard ball model [45],[46],[47]. In this model, however, collisions are elastic and reversible, whereas in our model they are inelastic and therefore irreversible. This allows the exchange of information between the interacting patterns. The corresponding computations are equally irreversible and therefore context-dependent. An additional essential difference with the billiard ball model is that computation in our model is naturally embedded in the ongoing behavior of a circuit.

Computation based on the propagations and interactions of coherent spiking patterns in neural systems is definitely a non-conventional form of computation. Conventional computation requires information to be represented and manipulated in the form of static symbols [27]. As the longstanding debate between computationalists and dynamicists [51] has pointed out, static symbols are less suitable to describe the temporal variability in the way the brain executes its functions and how it achieves flexibility. Dynamical computation can capture the spatiotemporal characteristics of brain activity patterns and provide them with an underlying computational interpretation. By synthesizing dynamics and computation, the present approach offers a starting point for a comprehensive understanding of the working mechanisms of the brain.

The collective propagation of activity patterns through a substrate of neurons can be portrayed as spatiotemporal spike chains. Our current emphasis on propagating patterns bears a similarity to the paradigm of synfire chains [52],[53], in which sequential spike chains play a central role. They are obtained by setting up feed-forward networks, designed to support wave-like spikes propagation through them. These networks perform information processing by synchronizing different spike chains [52],[53]. In our model, spatiotemporal spike chains are an emergent property of recurrent networks [54]. Rather than synchrony, their nonlinear pattern-forming capacities and transient interactions are the essential mechanisms for dynamical computation.

In the current study we have mainly focused on general-purpose computation based on ongoing, autonomous dynamics of neural circuits. We have also found that external perturbations can modulate the ongoing patterns, which include their propagations and interactions. Hence, propagating activity patterns could enable neural systems carry out some specific computations when actual sensory inputs are given. Indeed, propagating coherent patterns such as propagating waves have been found in evoked activity [7],[9],[11],[12],[14],[55]. Furthermore, during whole computing processes based on propagating coherent patterns, internal synaptic modifications and external feedbacks from other parts of the brain can be used to shape or control dynamical wave pattern to generate specific propagating patterns as required outputs or behavior sequences. The effect from feedback activity is analogous to use feedback signals to control waves patterns in spatially-extended non-equilibrium physical systems [56].

Instead of focusing on multiple, stationary patch patterns [57],[58] or single propagating wave pattern as in the most studies about neural fields [58],[59], the current model generates dynamical spiking activity patterns that can capture some of the complexities of empirically observed patterns. Therefore, the current study provides specific, experimentally testable predictions. In particular, the collective behavior of interacting, propagating coherent patterns belongs to the class of anomalous super-diffusion. As a process with underlying long-range coherence, collective anomalous super-diffusion is an important indicator of complicated, nontrivial interactions between propagating patterns. Its presence can be tested experimentally in a straightforward way. First qualitative indications that this process may occur in real neural circuits are the seeming randomness of the points of origin of neural activity patterns and the variability of their propagating directions [11],[22],[23]. More conclusive evidence can be obtained through calculating the MSD of the collective motions in the same way as for the current model data.

In the current dynamical computational framework, propagating coherent activity patterns are the fundamental primitives for signaling information and for processing information through their interactions. Indeed, at the level of neural circuits, signaling information by propagating coherent patterns has been clearly and very well documented as an important component of the function of the cortex [18]–[21]. Interactions between multiple active patterns, however, have merely been registered in experimental studies without considering their importance [11],[12],[13]. Our current work shows in an abstract, principled way how these interactions could play a key role in dynamical computation. For instance, in the visual cortex of ferrets, top-down influences have been found to be evident in terms of localized wave patterns [17], which could have collisions with wave patterns evoked by external visual inputs; such collisions might reflect “attention guided” processing of visual stimuli. It is, therefore, of crucial importance to study interactions between different propagating wave patterns experimentally and sow how they relate to the functions of the cortex.

## Methods

### 

#### A spiking neural circuit model

Our model represents biological neurons by integrate-and-fire spiking neurons that are uniformly distributed across a two-dimensional grid. The free dynamics of each neuron is:
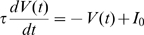
(2)where *t* is time, *V* is the membrane potential of the neuron, 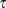 is the time scale of membrane potential change, 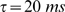, and 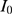 is constant external stimulation. Each neuron thus is an intrinsic oscillator. When the membrane potential reaches the threshold value 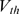 the neuron releases a spike, after which its membrane potential is reset and the neuron remains quiet for a refractory period 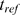. Each neuron receives excitatory and inhibitory inputs from other neurons. We include a delay time 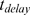 into the interactions between neurons. For simplicity, we chose 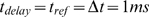; our results do not depend sensitively on these values. Considering the values of delay time and refractory time, we let the model evolve in time steps of 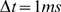. Eq. (2) can be integrated in 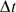 to obtain the membrane potential for a single neuron:
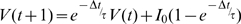
(3)Let 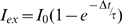, so Eq. (3) can be written as: 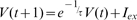. The whole spiking neural circuit is:
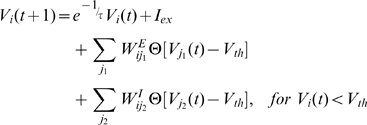
(4a)

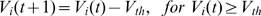
(4b)where 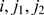 are indices of neurons, 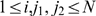. N is the total number of neurons, 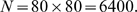 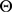 is the Heaviside step function: 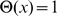 for 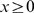, and 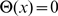 for 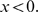 In the simple model, not all the details of real spiking behavior can be reproduced. To get spikes, the threshold 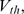 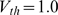 is used. The reason for using a threshold value is that for real neurons, after their membrane potentials reach certain threshold values, neuronal electrical activities are manifest as short electrical pulses (spikes) [60]. 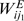 and 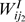 is the corresponding excitatory and inhibitory coupling strength from the 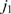
th and 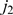
th neurons to the *i*th neuron. A similar model has been used to study the statistical properties of interspike intervals [61]. In our study, a “Mexican-hat” coupling scheme is used:
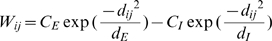
(5)where 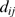 is the Euclidean distance between two neurons on a two-dimensional grid where the neurons occupy integer coordinates, and 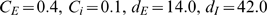. Connections between neurons are confined to 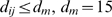, and periodic boundary conditions are used in the study. Some experimental studies have suggested that inhibitory connections are more spatially restricted than excitatory ones [62], and others have found that the opposite is true [63]. Based on these coupling parameters, in our model the range of excitatory connections is spatially more restricted than inhibitory ones.

For any *ith* neuron, from Eq. (5) we can obtain that when 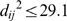, 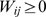, the input is excitatory and the neurons within this distance range are denoted as 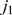 in Eq. (4a). The total excitatory synaptic input to the *ith* neuron is 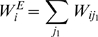; Otherwise when 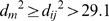, 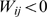, the input is inhibitory, and corresponding neurons in this range are denoted as 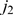 in Eq. (4a). The total inhibitory synaptic inputs to the *ith* neuron is 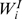, 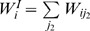. The excitatory coupling strength 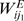 as used in Eq. (4) is 
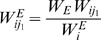
; the inhibitory coupling strength is 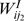, 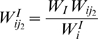. We used 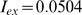. The parameter 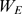 was varied within the range 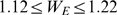, and 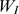 was varied within the range 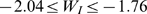 to obtain different spatiotemporal activity patterns. The network has been simulated with random initial conditions. Some initial time steps have been discarded until there is no significant change in the variability of interspike intervals of individual neurons.

#### Coherent and incoherent patterns

The patterns consist of the collective spiking activity of populations of neurons with clear spatial structures, i.e., spatially localized structures in our case. To quantify the spatial localization property, we used in our study the following criteria: (1) for a cluster of neurons that are firing, the distance between any two neurons within the cluster is smaller than 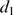, 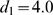; (2) the distance between the center-of-mass position of this cluster of neurons and that of any others is larger than 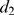, 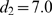. These criteria allow us to detect clustered patchy activity patterns that are spatially localized in terms of their spatial separation with others. Furthermore, we can discriminate coherent patterns and incoherent patterns, according to the statistics of their internal geometrical organizations at the level of individual spikes. The statistics can be obtained as follows. For a neuron within a given localized spiking pattern, we firstly draw a line to link its coordinate point where the neuron is located and the center-of-mass position point of the whole group, and then calculate the angle of the line related to the *x*-axis of the two-dimensional grid. We can get the angles for all neurons within the pattern. Then the angles are sorted into an ascending numerical order. For example, a pattern that has *j* neurons in total, after sorting, the series of the sorted angles is 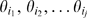, and the series of corresponding neurons is : 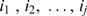. Then we calculate the distances between two successive neurons in this series, for instance the distance between the 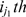 and the 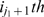 neuron is 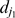. Finally, we can obtain the standard derivation of these distances, which is 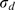. From the procedure used to get the standard derivation, it is apparent that this is a measure of the variability regarding how individual spikes are geometrically organized around the corresponding center-of-mass position; a smaller value means that the spikes are spatially more organized around its center-of-mass position. In our study we used the criterion that, a pattern is coherent when its 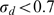, otherwise it is incoherent.

## Supporting Information

Figure S1 The mean value of the speeds of localized propagating patterns as a function of excitatory coupling strength.
(1.61 MB EPS)

Figure S2 The mean value of the sizes of localized propagating patterns as a function of excitatory coupling strength.
(1.15 MB EPS)

Video S1 The space-time behavior of two propagating coherent activity patterns. Among several self-sustained propagating patterns, for the purpose of revealing ongoing interactions between them, only two localized coherent structures are shown in the video.
(0.11 MB MOV)

Video S2 The ongoing behavior of several propagating, coherent activity patterns.
(0.19 MB MOV)

Video S3 The ongoing behavior of several propagating, coherent activity patterns.
(0.12 MB MOV)
